# The Bacterial Community Diversity of Bathroom Hot Tap Water Was Significantly Lower Than That of Cold Tap and Shower Water

**DOI:** 10.3389/fmicb.2021.625324

**Published:** 2021-04-23

**Authors:** Chiqian Zhang, Ke Qin, Ian Struewing, Helen Buse, Jorge Santo Domingo, Darren Lytle, Jingrang Lu

**Affiliations:** ^1^Pegasus Technical Services, Inc., Cincinnati, OH, United States; ^2^Oak Ridge Institute for Science and Education Participation Program, Office of Research and Development, United States Environmental Protection Agency, Cincinnati, OH, United States; ^3^Office of Research and Development, United States Environmental Protection Agency, Cincinnati, OH, United States

**Keywords:** drinking water, premise plumbing, community structure, community composition, temporal variation, 16S rRNA gene, high-throughput sequencing, public health

## Abstract

Microbial drinking water quality in premise plumbing systems (PPSs) strongly affects public health. Bacterial community structure is the essential aspect of microbial water quality. Studies have elucidated the microbial community structure in cold tap water, while the microbial community structures in hot tap and shower water are poorly understood. We sampled cold tap, hot tap, and shower water from a simulated PPS monthly for 16 consecutive months and assessed the bacterial community structures in those samples via high-throughput sequencing of bacterial 16S rRNA genes. The total relative abundance of the top five most abundant phyla (Proteobacteria, Actinobacteria, Bacteroidetes, Cyanobacteria, and Firmicutes) was greater than 90% among the 24 identified phyla. The most abundant families were Burkholderiaceae, Sphingomonadaceae, unclassified Alphaproteobacteria, unclassified Corynebacteriales, and Mycobacteriaceae. A multiple linear regression suggests that the bacterial community diversity increased with water temperature and the age of the simulated PPS, decreased with total chlorine residual concentration, and had a limited seasonal variation. The bacterial community in hot tap water had significantly lower Shannon and Inverse Simpson diversity indices (*p* < 0.05) and thus a much lower diversity than those in cold tap and shower water. The paradoxical results (i.e., diversity increased with water temperature, but hot tap water bacterial community was less diverse) were presumably because (1) other environmental factors made hot tap water bacterial community less diverse, (2) the diversity of bacterial communities in all types of water samples increased with water temperature, and (3) the first draw samples of hot tap water could have a comparable or even lower temperature than shower water samples and the second draw samples of cold tap water. In both a three-dimensional Non-metric multidimensional scaling ordination plot and a phylogenetic dendrogram, the samples of cold tap and shower water cluster and are separate from hot tap water samples (*p* < 0.05). In summary, the bacterial community in hot tap water in the simulated PPS had a distinct structure from and a much lower diversity than those in cold tap and shower water.

## Introduction

Municipal water utilities remove physiochemical contaminants from source water and inactivate microbes before discharging finished water to drinking water distribution systems (DWDSs) (Zhang and Liu, 2019). However, certain microbes, including opportunistic pathogens, survive disinfection and (re)grow in engineered water systems [EWSs, including DWDSs and building premise plumbing systems (PPSs)] (Hwang et al., 2012; Ashbolt, 2015a; El-Chakhtoura et al., 2018; Douterelo et al., 2019; Zhang and Liu, 2019; Zhang et al., 2019; Zhang and Lu, 2021). Microorganisms in EWSs corrode iron and steel pipes (Ren et al., 2015; Xing et al., 2018; Zhu et al., 2019), deteriorate water taste and color (Zhou et al., 2017; Boers et al., 2018; El-Chakhtoura et al., 2018), and threaten public health [e.g., water-related disease outbreaks due to the (re)growth of opportunistic pathogens] (Ashbolt, 2015b; Liu et al., 2017; Wang et al., 2017; El-Chakhtoura et al., 2018; Chen et al., 2019; Zhang and Lu, 2021). Each year, more than one dozen drinking-water-related disease outbreaks occur in the United States (US), and opportunistic pathogen *Legionella* (mainly from EWSs) causes over 50% of these outbreaks (Beer et al., 2015; Benedict et al., 2017). Other opportunistic pathogens such as non-tuberculous mycobacteria (NTM) and *Pseudomonas aeruginosa* also inhabit EWSs and potentially infect humans (Ashbolt, 2015b; Falkinham et al., 2015; Lu et al., 2016; Perrin et al., 2019).

Monitoring microbial community structures in EWSs is essential to estimate the health risks of water-related microbes (especially opportunistic pathogens), develop strategies to mitigate those risks, and meet increasingly strict drinking water regulations and policies (Hwang et al., 2012; Pinto et al., 2014; Wang et al., 2017; El-Chakhtoura et al., 2018; Perrin et al., 2019; Zhang and Liu, 2019; Zhang and Lu, 2021). Studies have intensely examined microbial community structures in cold tap/drinking water (as well as the corresponding biofilms) from EWSs and linked the structures to various factors such as seasonally changing environmental factors (e.g., ambient temperature, precipitation, and nutrients level) (McCoy and VanBriesen, 2012; Henne et al., 2013; Pinto et al., 2014; Ling et al., 2016; Prest et al., 2016b; Zhang and Liu, 2019; Vavourakis et al., 2020), treatment trains at water utilities (Pinto et al., 2012), disinfectant residual (e.g., free and combined chlorine) levels (Hwang et al., 2012; Waak et al., 2019; Zhang and Lu, 2021), and pipe network flushing (Douterelo et al., 2014; El-Chakhtoura et al., 2018). In addition to cold tap water, the end-consumers frequently contact two essential points of human exposure: hot tap water and shower water (a mixture of cold and hot tap water). In the field of water engineering, “hot tap water” refers to cold or normal municipal tap water that has been heated by a device (such as a local boiler and a mounted instant heater) either outside or within a PPS and reaches a temperature of 30°C or higher after the heating. Pathogenetic microbes in hot tap and shower water pose significant health risks (Henne et al., 2013; Lu et al., 2017). For instance, *Legionella*, an opportunistic pathogen causing legionellosis, is frequently present in hot tap water (Arnow et al., 1985; Farhat et al., 2012; Lesnik et al., 2016; Lu et al., 2017; Wolf-Baca and Siedlecka, 2019; Zhang and Lu, 2021). Shower water or showerhead is a reservoir for water-related *Mycobacterium avium* such as NTM, which infects human lungs (Nishiuchi et al., 2007, 2017; Falkinham et al., 2008; Feazel et al., 2009). However, research on microbial community structures in hot tap and shower water is scarce (Henne et al., 2013), and a comprehensive comparison of microbial community structures among cold tap, hot tap, and shower water is missing.

We recently conducted a long-term sampling campaign and assessed the population dynamics of representative opportunistic pathogens in cold tap, hot tap, and shower water from a home plumbing system (HPS) simulator (i.e., a simulated PPS) in a basement bathroom of a research building via quantitative polymerase chain reactions (qPCRs) (Lu et al., 2017). We concluded that water temperature caused the significant variations in the occurrence and abundance of those opportunistic pathogens. However, since qPCRs quantify only one group of microbes each time, we targeted only a few important opportunistic pathogens such as *Mycobacterium* spp., *Legionella* spp., and *P. aeruginosa*. Therefore, the structure of the whole bacterial community in the simulator was unknown.

We aimed to comprehensively understand the bacterial community structures in cold tap, hot tap, and shower water of the HPS simulator. We also aimed to reveal how environmental factors, such as the age of the simulator and disinfectant (i.e., free chlorine) residual concentration, affected the structures. We hypothesized that the bacterial community diversity was negatively correlated with disinfectant residual concentration because the residual would effectively suppress bacterial (re)growth (Zhang and Liu, 2019; Zhang and Lu, 2021). We also hypothesized that the diversity was positively correlated with the age of the simulator because the bacterial community of pipe biofilms and loose deposits with a greater diversity and density than that of the bulk water would detach from pipe surface when the simulator aged or the biofilms/deposits matured (Rittmann and McCarty, 2001; Henne et al., 2012; Liu et al., 2014, 2018; Petrova and Sauer, 2016; Prest et al., 2016a; Potgieter et al., 2018; Chan et al., 2019). We further hypothesized that the bacterial community in hot tap water was less diverse than those in cold tap and shower water because water in municipal EWSs typically has a relatively low temperature (i.e., < 25 or 30°C) (Blokker and Pieterse-Quirijns, 2013; Eck et al., 2016; Zlatanovic et al., 2017; Agudelo-Vera et al., 2020) and only a small percentage of drinking water bacteria could adapt to the high temperature (i.e., > 30°C) of hot tap water. Therefore, we also compared the bacterial communities in cold tap, hot tap, and shower water to more comprehensively understand their structures in the simulator. This work provides deep insights into how environmental factors affect water bacterial community structures in PPSs and the interrelationship among cold tap, hot tap, and shower water bacterial communities.

## Materials and Methods

### Study Location and Water Sampling

We collected cold tap, hot tap, and shower water samples from the HPS simulator constructed for research purposes in a basement bathroom of a research building in a major city in the East North Central region of the Midwestern US (Cahalan and Lytle, 2017; Lytle et al., 2018, 2021). Cold tap water of the building PPS was directly pumped to the HPS simulator. The building PPS was fed with the water from a typical municipal water utility. The utility used river water as source water and sequentially conducted coagulation, flocculation, sedimentation, sand and gravel filtration, granular activated carbon filtration, and chlorination before discharging the finished water to a typical municipal DWDS (Gomez-Alvarez et al., 2015; Buse et al., 2019). The typical source water, treatment train at the water utility, and structure of the DWDS ensured that the water samples analyzed in this study were representative municipal drinking water of the Midwestern US.

The HPS simulator contained approximately 56 m Type M copper pipes (inside diameter 1.45 cm), a flow meter totalizer, a 454-L GE^®^ electric water heater (Model GE40M06AAG, General Electric Company, Boston, Massachusetts, United States), a Glacier Bay showerhead (Model 875-2101, The Home Depot, Inc., Atlanta, Georgia, United States), and four faucets. The flow meter was at the start of the simulator and recorded the flow rate of the whole simulator. The water heater at the start of the hot water recirculation loop generated hot water (temperature set at 49°C). Faucet 1 was a cast brass utility faucet. Faucets 2 to 4 were three identical bathroom-type hot-and-cold water faucets (i.e., mixing faucets; Model 8125F, American Standard, Piscataway, New Jersey, United States) with a chrome exterior and a cast brass and plastic interior. The lengths of the cold water line from the flow meter to Faucet 1, Faucet 2, Faucet 3, Faucet 4, and the showerhead were 16.6, 14.8, 12.0, 9.1, and 15.5 m, respectively. The lengths of the hot water line from the electric water heater to Faucet 1, Faucet 2, Faucet 3, Faucet 4, and the showerhead were 18.4, 16.1, 13.2, 10.2, and 16.4 m, respectively. Other important HPS simulator components included brass ball valves, brass check valves, a bathtub, and a toilet.

The HPS simulator was constructed on January 23, 2012, and was operated only on weekdays. We operated the HPS simulator under a “controlled-use” flushing schedule to simulate the average daily water use for a typical household of four residents (target total daily water use 708 L). We achieved this target water use by manually flushing all four faucets three times per weekday at 8:00 AM (cold tap water only), 12:00 PM (a 50:50 blend of cold and hot tap water), and 3:00 PM (cold tap water only). The toilet and showerhead were flushed with cold tap water and a 50:50 blend of cold and hot tap water, respectively, each weekday at 8:00 AM, 12:00 PM, and 3:00 PM. Faucet 1, Faucet 2, Faucet 3, Faucet 4, and the showerhead were flushed for 7, 7, 15, 1, and 15 min each time, respectively.

Our recent work detailed the water sampling (Lu et al., 2017). Briefly, we sampled water monthly (except May 2013) from March 2012 to July 2013 from Faucet 4 (for cold and hot tap water) and the showerhead (for shower water) (16 sampling events, 32 one-liter samples for each water type, 96 samples in total) (Supplementary Table 1 in Supplementary material). On each sampling day (around 7 AM on Wednesday and occasionally Tuesday or Thursday), we collected the first draws of cold and hot tap water immediately after turning the taps on. We then collected the second draws of cold and hot tap water after running the taps for 3 min. After collecting the tap water samples, we flushed the showerhead for 3 min and subsequently collected two consecutive shower water (a blend of cold and hot tap water) samples (i.e., the first and second draws). The volumes of stagnant cold and hot tap water for Faucet 4 were approximately 1.49 and 1.67 L, respectively. The volumes of stagnant cold and hot tap water for the showerhead were approximately 2.55 and 2.68 L, respectively. Running faucets/taps and showerheads for minutes (or even seconds) before sampling to release stagnant water and get representative water samples is a widely accepted standard practice in water engineering (Zacheus and Martikainen, 1994; Leoni et al., 2005; Bargellini et al., 2011; Wang et al., 2012; Henne et al., 2013; Serrano-Suárez et al., 2013; Bukh and Roslev, 2014; Donohue et al., 2014, 2019a,b; Wolf-Baca and Siedlecka, 2019; Isaac and Sherchan, 2020). Because the faucet (i.e., Faucet 4) and showerhead were flushed for a sufficient amount of time prior to the sampling of shower water, the first draw samples of shower water were already representative samples. Therefore, unlike sampling tap water, we did not flush the showerhead between sampling the two draws of shower water but collected the two draws of shower water consecutively on each sampling day.

After collecting each water sample (1 L) in a sterile flask, we immediately measured water temperature and chlorine (free and total) residual concentrations (Supplementary Table 1). We determined free and total chlorine residual concentrations with US Environmental Protection Agency (US EPA) approved Methods 10231 and 10232, respectively, using a Free and Total Chlorine TNTplus^®^ Vial Test kit (TNT867, limit of detection 0.05 mg Cl_2_⋅L^–1^; Hach Company, Loveland, Colorado, United States). We recorded the absolute sampling time (representing the age of the simulator) for each sampling day (e.g., the samples of March 2012 and July 2013 were 0 and 484 d samples, respectively). In this work, “age” and “aged” specifically apply to the newly built simulator in the bathroom. We also collected the daily average ambient temperature data of the city where the research building is located in for each sampling day from the National Weather Service (National Oceanic and Atmospheric Administration, US Department of Commerce; weather.gov) (Supplementary Table 2).

### Total Genomic DNA Extraction and Quantification

We extracted total genomic DNA from water samples following an established procedure (Lu et al., 2016, 2017). Briefly, we filtered each entire water sample (1 L) with a 0.4 μm pore size polycarbonate membrane to capture microbial cells. The membrane was transferred to a Lysing Matrix A Tube (MP Biomedicals, Santa Ana, California, United States) containing garnet powder and a ceramic sphere. The tube was then stored at −80°C until DNA extraction.

To extract DNA from the stored cells, we added 400 μL of 1× Tissue and Cell Lysis Solution (Epicenter Technologies Corp., Madison, Wisconsin, United States) to each tube. We then shook each tube with a Mini-Beadbeater-16 (BioSpec Products, Inc., Bartlesville, Oklahoma, United States) to lyse the cells. We subsequently centrifuged each tube and recovered total genomic DNA from the supernatant using a MasterPure™ Complete DNA and RNA Purification Kit (Epicenter Technologies Corp., Madison, Wisconsin, United States). A final 100 μL of DNA solution was collected in a microcentrifuge tube for each water sample. We determined the concentration and purity of each DNA sample with a Nanodrop™ 1000 Spectrophotometer (Thermo Scientific, Wilmington, Delaware, United States). The DNA samples were stored at −80°C until use.

### High-Throughput Sequencing of Bacterial 16S rRNA Genes

We amplified the V4 variable region of bacterial 16S rRNA genes from the DNA samples by PCRs (one PCR with one primer set for each DNA sample) using a TaKaRa Ex Taq^®^ DNA Polymerase Kit (Takara Bio United States, Inc., Mountain View, California, United States). The forward primer 515F (5′-GTG CCA GCM GCC GCG GTAA-3′) was identical for all PCRs. However, each PCR had a unique reverse primer consisting of 806R (5′-GGA CTA CHV GGG TWT CTA AT-3′) and a sample-specific Golay barcode (Caporaso et al., 2012; Walters et al., 2016). Each PCR totaled 25 μL with 200 nM each (forward and reverse) primer and 2 μL of DNA sample (equivalent to 20 mL of original water sample). The PCR thermal cycling conditions were 5 min at 94°C, 30 cycles of (45 s at 94°C, 60 s at 50°C, and 90 s at 72°C), and 10 min at 72°C (Qin et al., 2017). After checking the quality of the PCR amplicons with agarose gel electrophoresis, we pooled and purified the amplicons of different DNA samples. The purified amplicons were paired-end sequenced (2 × 250 bp) on an Illumina MiSeq platform (Illumina, Inc., San Diego, California, United States) at the DNA Core Facility of Cincinnati Children’s Hospital (Cincinnati, Ohio, United States).

### Data Availability Statement

The raw sequencing data are available at the National Center for Biotechnology Information’s Sequence Read Archive (SRA) website (ncbi.nlm.nih.gov/sra) (BioProject ID: PRJNA598369; Tax ID: 2651591; SRA IDs: SRR10810816 to SRR10810911; Accession numbers: SAMN13704937 to SAMN13705032).

### Sequencing Data Cleaning and Contig Alignment

We used Mothur (version 1.43.0) (Schloss et al., 2009; López-García et al., 2018; Schloss, 2020) to analyze the sequencing data following a standard procedure (mothur.org/wiki/MiSeq_SOP; accessed December 2019) (Kozich et al., 2013) and two extended protocols (Batut et al., 2018; Hiltemann et al., 2018; Chappidi et al., 2019). The raw paired-end reads were merged to 2,838,570 contigs. The contigs having more than two mismatches with 515F or 806R were removed. We trimmed the remaining contigs to remove the sequences of the primers. We then removed the contigs with any ambiguities or longer than 275 bp and created a contig dataset containing 2,284,563 contigs (339,300 unique contigs).

We used a Mothur-compatible (i.e., customized) SILVA database (full length version, release 132) (Quast et al., 2012; Yilmaz et al., 2013; López-García et al., 2018) as the reference database. The coordinates for 515F and 806R in the original (i.e., un-customized) SILVA database are 13,862 and 23,444, respectively (Ben Guerrero et al., 2016; Sapountzis et al., 2019). To customize the database, we removed the sequences before position 13,862, the sequences after position 23,444, the leading dots, and the trailing dots from the original SILVA database. The customized reference database was 9,582 columns wide.

We aligned the 2,284,563 contigs to the customized reference database and found that more than 95% of the 2,284,563 contigs started at position 8 and ended at position 9,581 or 9,582 of the customized database. We then removed the contigs that started after position 8, ended before position 9,581, or had a stretch of more than eight repeated bases from the alignment (i.e., the contig dataset aligned to the customized database). The resultant alignment had 2,210,140 contigs (321,196 unique contigs). We further removed any overhangs on either end of the V4 region of bacterial 16S rRNA genes and columns with only gap characters (“-” or “.”) from the alignment. The removal of overhangs and gap characters reduced the size of the alignment from 9,582 to 521 columns wide and created 17,964 duplicate contigs. Therefore, we merged the contigs with the same sequences and created an alignment with 303,232 unique contigs (2,210,140 contigs). We further merged the unique contigs with one or two nucleotide differences (pre-clustering), generating an alignment with 103,321 unique contigs (2,210,140 contigs). We also removed chimerical contigs using VSEARCH (version 2.13.3, implemented in Mothur) (Rognes et al., 2016) and obtained an alignment with 2,119,850 contigs (71,056 unique contigs; 4.1% of the 2,210,140 contigs were chimerical). We classified the unique contigs using a naïve Bayesian classifier (Wang et al., 2007) and the original (i.e., un-customized) SILVA database (cutoff 80%). Contigs belong to Eukaryota, chloroplasts, mitochondria, and Arachaea as well as those could not be classified to a domain were removed. The resultant alignment had 2,104,926 contigs (69,747 unique contigs).

### Taxonomic Classification

We calculated the uncorrected pairwise distances between aligned contigs with a one-gap calculator (Sogin et al., 2006; Schloss, 2010) and obtained 114,076,573 pairs of contigs (each pair had a distance less than 0.03). We then identified 3,821 operational taxonomic units (OTUs) from the aligned contigs using the OptiClust algorithm (cutoff 0.03) (Westcott and Schloss, 2017). We also counted the contigs in each OTU for each water sample (Supplementary Table 3) and identified the taxonomy for each OTU (Supplementary Table 4).

### Bacterial Community Structure Examination

We assessed the alpha diversity of the bacterial communities in the water samples by generating rarefaction curves with the Chao1 estimator (Chao, 1984; Colwell and Coddington, 1994; Schloss et al., 2009; Chao and Shen, 2010; Eren et al., 2012) (Supplementary Table 5 and Supplementary Figure 1). We also calculated the Shannon diversity index (proportional to both community richness and evenness) (Shannon, 1948; Chao and Shen, 2003; Spellerberg and Fedor, 2003; Grice et al., 2009; Lu et al., 2012; Waak et al., 2019) (Supplementary Figure 2) and the Inverse Simpson diversity index (proportional to both community richness and evenness) (Simpson, 1949; Li et al., 2012; Blasiak et al., 2014) (Supplementary Figure 3) for the bacterial community in each water sample. Richness and evenness are the two key components for community diversity (Zhang et al., 2012).

To analyze the beta diversity of the bacterial communities, we first calculated the numbers of OTUs shared by two groups of water samples with the Observed-richness calculator (Schloss et al., 2009; Schloss, 2020). We then determined which shared OTUs had significantly different abundance between the two groups with the Metastats program (White et al., 2009). We also calculated the Yue and Clayton theta similarity coefficients (to indicate the dissimilarity between the structures of communities) (Yue and Clayton, 2005) and the classical Jaccard similarity coefficients (to indicate the dissimilarity between the membership of communities) (Jaccard, 1908, 1912). The Jaccard index was chosen because it is the most widely used one among all similarity indices (Yue and Clayton, 2005). The Yue and Clayton distance matrix was chosen because of its frequent use in community similarity analysis and capability to distinguish between a population and a sample (Koyano et al., 2014; Ly et al., 2018; Paredes-Montero et al., 2020; Tal et al., 2020; Trudeau et al., 2020). Those two distance matrices were analyzed with a three-dimensional Principal coordinates analysis (3D PCoA) and a 3D Non-metric multidimensional scaling (3D NMDS). The PCoA and NMDS better explain the Yue and Clayton theta distance matrix than explain the Jaccard distance matrix (Supplementary Table 6). Therefore, only the Yue and Clayton theta distance matrix was analyzed hereinafter. Next, to determine whether the bacterial community diversity between two or among three groups of water samples was homogeneous (i.e., whether the variations of different groups were distinct), we conducted a Homogeneity of molecular variance test (HOMOVA test; a non-parametric analog of the Bartlett’s test) (Bartlett, 1937; Stewart and Excoffier, 1996; Schloss, 2008). Using an Analysis of molecular variance test (AMOVA test; a non-parametric analog of Analysis of variance) (Excoffier et al., 1992; Martin, 2002; Schloss, 2008), we further tested whether the points representing samples in different groups of water samples in the Yue and Clayton theta distance matrix have significantly different centroids.

We ranked the 3,821 OTUs according to their significance in shifting the water samples along the three axes of the 3D NMDS ordination by calculating the Spearman’s rank correlation coefficients, which indicate the correlations between the relative abundance of OTUs and the axes (Zar, 1972; Gauthier, 2001; Hauke and Kossowski, 2011; Mukaka, 2012; Tkachuk et al., 2014). We also used the Spearman’s rank correlation coefficients to determine whether water temperature, absolute sampling time (i.e., the age of the HPS simulator), total chlorine residual concentration, and ambient temperature significantly moved the water samples in the 3D NMDS ordination. In addition, using the Dirchlet-multinomial mixture (DMM) model (Holmes et al., 2012) and the Square root of the Jensen-Shannon divergence calculator (Endres and Schindelin, 2003; Osterreicher and Vajda, 2003; Fuglede and Topsoe, 2004), we tested whether the bacterial communities in the water samples can be partitioned to separate metacommunities or enterotypes.

We converted the phylip-formated Yue and Clayton theta distance matrix to a Newick-formatted dendrogram using the unweighted pair-group method with an arithmetic mean algorithm (Sokal and Michener, 1958; Huelsenbeck and Kirkpatrick, 1996). The dendrogram describes the dissimilarity (i.e., one minus the similarity) among the bacterial communities in cold tap, hot tap, and shower water. The dendrogram was visualized with a FigTree software (version 1.4.4) (Rambaut, 2018). We tested whether the clustering of different groups of water samples in the dendrogram is statistically significant using three assays: (1) the parsimony approach (the branch length of the dendrogram ignored) (Slatkin and Maddison, 1989, 1990; Maddison and Slatkin, 1991; Schloss and Handelsman, 2006), (2) the unweighted UniFrac algorithm (the branch length incorporated; the weightings uncorrected), and (3) the weighted UniFrac algorithm (the branch length incorporated; the weightings corrected) (Lozupone and Knight, 2005; Lozupone et al., 2007; Schloss, 2008).

### Statistical Analysis, Linear Regression, and Figure Plotting

We performed statistical analysis with Mothur (version 1.43.0) (Schloss et al., 2009; Schloss, 2020) or an SPSS^®^ Statistics software (version 26.0, International Business Machines Corporation, Armonk, New York, United States) (Landau and Everitt, 2003; Elliott and Woodward, 2007; Gaur and Gaur, 2009). Means ± standard deviations are reported. The significance level is 0.05. We conducted linear regression using the SPSS^®^ Statistics or Microsoft Office 365 ProPlus Excel (version 1902, Microsoft Corporation, Redmond, Washington, United States). Figures were plotted with Excel unless specified otherwise.

## Results and Discussion

### Correlations Among Water Temperature, Ambient Temperature, and Chlorine Residual Concentration

The ambient temperature and the temperature for cold tap, hot tap, and shower water were normally distributed (the Shapiro-Wilk test with SPSS^®^, *p* > 0.05) (Supplementary Table 7). The temperature of the first draw of cold tap water had a negative linear correlation with ambient temperature (*R*^2^ 0.253, *p* 0.047) (Supplementary Figure 4). However, this correlation has only a marginal *p*-value close to 0.05 (i.e., the selected significance level) and a relatively small *R*-squared value, indicating that the ambient temperature had a limited effect on the temperature of the first draw of cold tap water. Similarly, the temperature of the second draw of cold tap water lacked a linear correlation with ambient temperature (*R*^2^ 0.060, *p* 0.362) (Supplementary Figure 4). Therefore, the temperature of cold tap water (both first and second draws) was insensitive to the changes in ambient temperature. In addition, the temperature of cold tap water had limited seasonal variations (Supplementary Table 1). In summary, the temperature of cold tap water (both first and second draws) did not vary significantly over the 16-month sampling campaign (i.e., relatively stable over time).

The mean temperatures for the first draws of cold (20.9 ± 1.1°C) and hot (30.4 ± 5.3°C) tap water were much lower than those of the second draws (30.1 ± 3.3°C and 46.9 ± 1.2°C for cold and hot tap water, respectively) (Supplementary Table 1). The cold tap water in the PPS of the research building is warmer than that in the HPS simulator. The first draw of cold tap water was stagnant water sitting in the cold water line in the simulator overnight (the last flushing before the sampling occurred at 3:00 PM the day before), and the second draw of cold tap water was representative water from the cold water line of the building plumbing. Similarly, the first draw of hot tap water was stagnant water, while the second draw of hot tap water was representative water from the electric water heater because its temperature was close to the set temperature of the heater (49°C). The temperatures for cold and hot tap water significantly predict shower water temperature (multiple linear regression with SPSS^®^, *R*^2^ 0.513, *p* < 0.001) because shower water was a mixture of cold and hot tap water. The concentrations of free and total chlorine residuals had a strong positive linear correlation (*R*^2^ 0.986, *p* < 0.001) (Supplementary Figure 5). Therefore, we used total chlorine residual to represent disinfectant residuals hereinafter. Total chlorine residual concentration (0.11 ± 0.12 mg Cl_2_⋅L^–1^, *n* = 96) lacked a significant linear correlation with water temperature (19.0 to 49.0°C, 34.2 ± 8.8°C, *n* = 96) (*p* 0.598) (Supplementary Figure 6).

### The Bacterial Community Compositions of Cold Tap, Hot Tap, and Shower Water Were Distinct

After cleaning the sequencing data, we obtained 69,747 unique bacterial 16S rRNA gene sequences (i.e., the contigs) representing 2,104,926 contigs from the 96 water samples. The number of contigs per water sample varied from 12,291 to 34,753 (21,926 ± 4,674 contigs per sample) (Supplementary Table 3). We identified 3,821 OTUs from those 2,104,926 contigs (Supplementary Table 4). We further classified 24 phyla (Supplementary Table 8) and 248 families (Supplementary Table 9) from those 3,821 OTUs.

**FIGURE 1:**
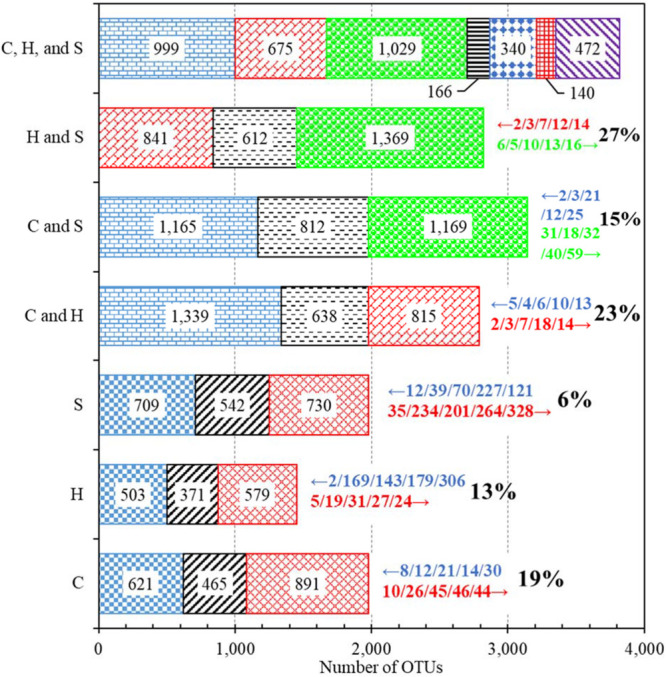
Numbers of OTUs identified from different groups of water samples. **C**: Cold tap water. **H**: Hot tap water. **S**: Shower water. For each of the three horizontal bars of **C**, **H**, and **S**, the left, middle, and right segments represent the numbers of OTUs unique to the first draw, shared by the two draws, and unique to the second draw, respectively. For each of the three bars of **C and H**, **C and S**, and **H and S**, the left, middle, and right segments represent the numbers of OTUs unique to cold (for the bars of **C and H** and **C and S**) or hot (for the bar of **H and S**) tap water, shared by the two groups of water samples, and unique to hot tap (for the bar of **C and H**) or shower (for the bars of **C and S** and **H and S**) water, respectively. For the bar of **C, H, and S**, the first, second, third, fourth, fifth, sixth, and seventh (left to right) segments represent the numbers of OTUs unique to cold tap water, unique to hot tap water, unique to shower water, shared by cold and hot tap water (excluding shower water), shared by cold tap and shower water (excluding hot tap water), shared by hot tap and shower water (excluding cold water), and shared by cold tap, hot tap, and shower water, respectively. Numbers within bar segments: Numbers of OTUs. Numbers marked with leftward and rightward arrows immediately next to the bars (on the right): The IDs of the top five most important OTUs (descending order of abundance, left to right) that had significantly greater (*p* < 0.05) abundance in the groups represented by the farleft and farright segments, respectively. Percents next to the rightward arrows (on the right): Percents of OTUs (among the shared ones) that had significantly different abundance between the two groups (*p* < 0.05).

The bacterial communities of the first and second draws of any water type (i.e., cold tap, hot tap, and shower water) shared 542 or less OTUs (Figure 1). For any water type, most (57% or more) of the OTUs for any draw were unique to that draw, suggesting that the two draws had different bacterial community compositions. Only 6% of the OTUs shared by the two draws of shower water had significantly different abundance between the two draws, while the figures for cold (19%) and hot (13%) tap water were much greater. The small percent (6%) for shower water was due to the consecutive sampling strategy. The relatively large precents for cold and hot tap water (19% and 13%, respectively) were because that the first draws were stagnant water and the second draws were representative water.

The bacterial communities of cold tap, hot tap, and shower water had 3,821 OTUs in total but shared only 472 OTUs (12.4% or 472/3,821) (Figure 1). We identified 15 phyla from those 472 shared OTUs. Proteobacteria (relative abundance 65.9% or 311/472), Actinobacteria (relative abundance 13.1% or 62/472), and Bacteroidetes (relative abundance 5.5% or 26/472) were the dominant phyla (Supplementary Table 10). Any two groups of samples (i.e., cold and hot tap water, cold tap and shower water, and hot tap and shower water) shared 812 or less OTUs (Figure 1). Thus, most (56% or more) of the OTUs for each group of samples (i.e., cold tap, hot tap, and shower water) were unique to that group. Therefore, those three groups of water samples from the same HPS simulator had different bacterial community compositions.

This study found that the first and second draws for either cold or hot tap water sampled within a short time from the same faucet had distinct bacterial community compositions (Figure 1). This distinction suggests that the bacterial community composition for stagnant tap water changed significantly overnight (the duration between sampling and the last flushing the day before was approximately 16 h). In addition, any two types of water (i.e., cold tap, hot tap, and shower water) from nearby plumbing endpoints had distinct bacterial community compositions, indicating water temperature and other factors dramatically altered the compositions. The rarefaction curves for some water samples do not level off (data not shown), suggesting that a deeper sequencing would have identified more OTUs from those samples (Liao et al., 2013). However, the rarefaction curves for the first and second draws of any water type (i.e., rarefaction curves for “pooled” water samples) do generally level off (Supplementary Figure 1), indicating that the dominant species or OTUs for the first and second draws had already been sampled at the current sequencing depth. Therefore, the distinction in bacterial community compositions would not significantly change with a greater sequencing depth.

Fifteen percent to 27% of the OTUs shared by any two groups of water samples (i.e., cold tap, hot tap, and shower water) had significantly different abundance (Figure 1). Therefore, most (> 70%) of the OTUs shared by any two groups had comparable abundance. The underlying reason could be that all water samples were originated from the same cold tap water of the nearby building plumbing. In addition, the percent of OTUs with significantly different abundance between cold tap and shower water (15% of the shared OTUs) was obviously lower than those between cold and hot tap water (23% of the shared OTUs) and between hot tap and shower water (27% of the shared OTUs). As a result, the bacterial community structure of hot tap water could be different from those of cold tap and shower water.

**FIGURE 2:**
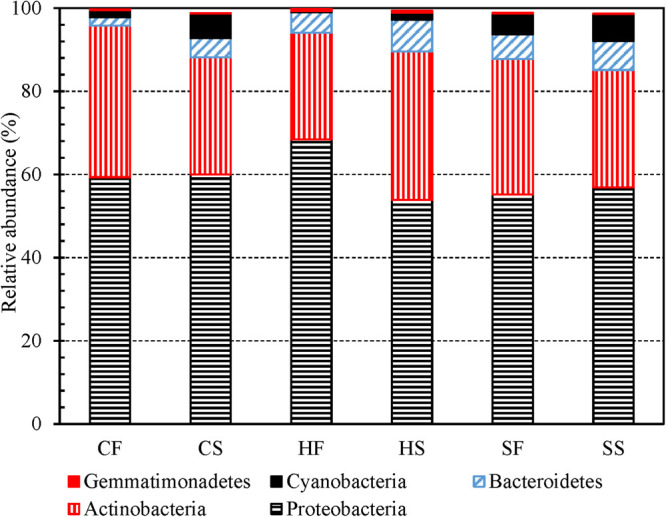
The relative abundance of dominant bacterial phyla in the first and second draws of cold tap, hot tap, and shower water (six groups of water samples). **CF**: Cold tap water-First draw. **CS**: Cold tap water-Second draw. **HF**: Hot tap water-First draw. **HS**: Hot tap water-Second draw. **SF**: Shower water-First draw. **SS**: Shower water-Second draw. We assigned a phylum to each contig for each water sample with Mothur and then calculated the relative abundance of each phlyum for each group of samples. We displayed a phylum only if its relative abundance is greater than 0.50% in at least one group.

Proteobacteria, Actinobacteria, Bacteroidetes, Cyanobacteria, and Firmicutes (descending order of the number of OTUs in a phylum) were the top five most abundant phyla identified from the 3,821 OTUs (Supplementary Table 8). Previous studies also classified Proteobacteria, Actinobacteria, Cyanobacteria, and Bacteroidetes to be the most abundant phyla in drinking water (Santo Domingo et al., 2003; Williams et al., 2004; Revetta et al., 2010, 2011; Zhang and He, 2013; Wang et al., 2018). For any water type, the first and second draws had similar compositions at the phylum level (Figure 2). In addition, Proteobacteria, Actinobacteria, Bacteroidetes, and Gemmatimonadetes (dominant phyla) had similar relative abundance in cold tap, hot tap, and shower water (Supplementary Figure 7A). Furthermore, the relative abundance of dominant phyla in the first and second draws for any water type changed following similar trends over the 16-month sampling period (Supplementary Figures 7B to 7G). Likewise, the changes in the relative abundance of dominant phyla for cold tap, hot tap, and shower water over the 16-month sampling period followed similar trends (Supplementary Figures 7B to 7G). Therefore, cold tap, hot tap, and shower water had comparable bacterial community compositions at the phylum level.

The relative abundance of Actinobacteria (class) in the first draw of cold tap water decreased over the 16-month sampling period (Supplementary Figure 8A). However, the relative abundance of Actinobacteria in the second draw of cold tap water decreased but subsequently increased over time (Supplementary Figure 8B). Actinobacteria in the first draw of hot tap water reached its maximum relative abundance in August 2012 (Supplementary Figure 8C), but its maximum relative abundance in the second draw of hot tap water occurred in April 2012 (Supplementary Figure 8D). The first and second draws for either cold or hot tap water were stagnant and representative water, respectively. Therefore, the bacterial community compositions at the class level for stagnant and representative water were distinct. For shower water, since the two draws on each sampling day were consecutive samples, the relative abundance of dominant classes for those two draws changed over time following similar trends (Supplementary Figures 8E and 8F). Furthermore, the relative abundance for dominant classes in cold tap, hot tap, and shower water changed over the 16-month sampling period following distinct trends (Supplementary Figure 8).

**FIGURE 3:**
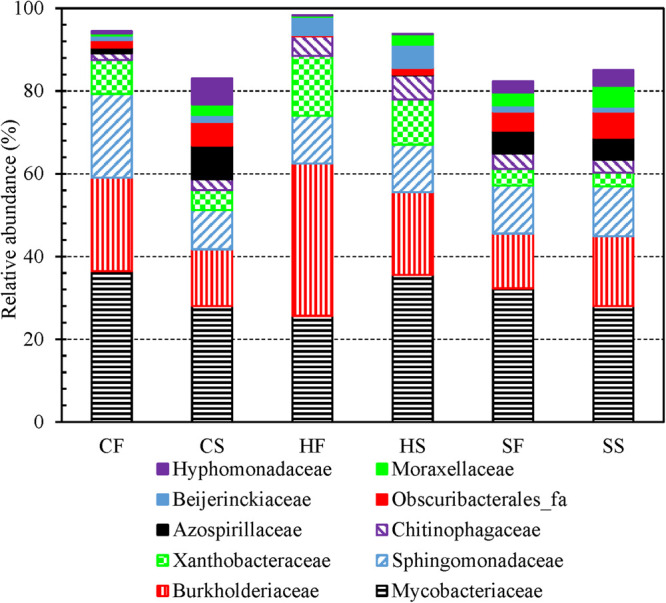
The relative abundance of dominant bacterial families in the first and second draws of cold tap, hot tap, and shower water (six groups of water samples). **CF**: Cold tap water-First draw. **CS**: Cold tap water-Second draw. **HF**: Hot tap water-First draw. **HS**: Hot tap water-Second draw. **SF**: Shower water-First draw. **SS**: Shower water-Second draw. We assigned a family to each contig for each water sample with Mothur and then calculated the relative abundance of each family for each group of samples. We displayed a family only if its relative abundance is greater than 5.00% in at least one group.

The top five most abundant families identified from the 3,821 OTUs were Burkholderiaceae, Sphingomonadaceae, unclassified Alphaproteobacteria, unclassified Corynebacteriales, and Mycobacteriaceae (descending order of number of OTUs in a family) (Supplementary Table 9). Mycobacteriaceae contains a single genus *Mycobacterium* (Lory, 2014) which is an important drinking water opportunistic pathogen (Good, 1985; Marciano-Cabral et al., 2010; Lu et al., 2017; Wang et al., 2019; Zhang and Lu, 2021). For both cold and hot tap water, the relative abundance of Burkholderiaceae (one dominant family in drinking water and PPSs) (Zeng et al., 2013; Buse et al., 2014; Ferro et al., 2019; Vavourakis et al., 2020) and Xanthobacteraceae (associated with nitrogen fixation) (Oren, 2014) obviously dropped in the second draws (compared with the first draws) (Figure 3). By contrast, the relative abundance of Azospirillaceae (associated with nitrogen fixation) (Sridevi et al., 2012), Obscuribacterales_fa, Moraxellaceae (contains potentially opportunistic pathogens) (Pettersson et al., 1998; Inkinen et al., 2018), and Hyphomonadaceae (contains “strict aerobic and stalked and non-stalked species”) (Abraham and Rohde, 2014) significantly increased in the second draws for both cold and hot tap water. The relative abundance of Mycobacteriaceae dropped in the second draw of cold tap water but increased in the second draw of hot tap water. Sphingomonadaceae (strictly aerobic chemoheterotrophs; a reservoir of antimicrobial resistance in drinking water) (Vaz-Moreira et al., 2011) had a significantly lower abundance in the second draw than in the first draw of cold tap water but a comparable abundance for the two draws of hot tap water. The relative abundance of Chitinophagaceae (aerobic or facultative anaerobic) (Cao et al., 2017) slightly increased in the second draws for both cold and hot tap water. For shower water, since the two draws on each sampling day were consecutive samples, they had comparable bacterial community compositions at the family level (Figure 3).

The relative abundance of Burkholderiaceae and Xanthobacteraceae (families) decreased from hot tap water, to cold tap water, and to shower water (Supplementary Figure 9). The relative abundance of Chitinophagaceae (family) decreased from hot tap water, to shower water, and to cold tap water. By contrast, the relative abundance of Obscuribacterales_fa and Azospirillaceae (families) in hot tap water was much lower than that in cold tap and shower water. The relative abundance of Mycobacteriaceae (approximately 30%) and Sphingomonadaceae (12% to 15%) (families) were similar across cold tap, hot tap, and shower water. As a result, the bacterial community compositions of cold tap, hot tap, and shower water at the family level were distinct.

In conclusion, at the phylum level, the first and second draws for any water type had similar bacterial community compositions, while cold tap, hot tap, and shower water also had comparable compositions (Figure 2 and Supplementary Figure 7). At the class and family levels, the first and second draws of both cold and hot tap water had distinct bacterial community compositions (Figure 3 and Supplementary Figure 8) because the first draws were stagnant water and the second draws were representative water. By contrast, the compositions for the two draws of shower water were similar at the class and family levels because those two draws were consecutive samples. In addition, cold tap, hot tap, and shower water had distinct bacterial community compositions at the class and family levels (Supplementary Figures 8 and 9). In the HPS simulator, cold tap water was the direct and sole source of hot tap water, and shower water was a mixture of cold and hot tap water. Moreover, the cold tap, hot tap, and showerhead were close to one another in the same simulator where all water pipes had the same size, material, and age. Therefore, the distinct bacterial community compositions for cold tap, hot tap, and shower water at the class and family levels suggest that different endpoints of the simulator had distinct physicochemical and microbial conditions. Those biotic and abiotic factors significantly affected drinking water bacterial community composition. For instance, biofilms and loose deposits on water pipes, faucets, and showerheads are an important source of microbes of the corresponding bulk water and significantly affect the bulk water microbial communities (Henne et al., 2012; Liu et al., 2014, 2018; Petrova and Sauer, 2016; Prest et al., 2016a; Potgieter et al., 2018; Chan et al., 2019). The biofilms and loose deposits adapted to the local environment of the cold water line, cold tap, hot water line, hot tap, and showerhead in the simulator would release unique bacterial species to the bulk water (Bollin et al., 1985; Feazel et al., 2009; Lu et al., 2017), contributing to the distinct community compositions of cold tap, hot tap, and shower water at the class and family levels. Furthermore, the bacterial consortium would have a fast transformation when the electric water heater produced hot tap water by heating cold tap water (Henne et al., 2013). When cold tap water was heated, certain bacteria survived (i.e., the carryover), many bacterial species decayed, and some high-temperature-tolerant (i.e., thermophilic) bacteria rapidly outgrew other species. Therefore, the bacterial community compositions for hot tap and shower water were significantly different from that of cold tap water at the class and family levels.

At the phylum (Figure 2 and Supplementary Figure 7A), class (Supplementary Figure 8), and family (Figure 3 and Supplementary Figure 9) levels, the cumulative relative abundance for the dominant groups of bacteria was greater than 70%. A Krona plot (Ondov et al., 2011) of the community composition based on the taxonomy of the contigs for all water samples also indicates that a few dominant groups of bacteria represented the majority of the whole community (Supplementary Figure 10). As a result, the bacterial community in the HPS simulator was uneven.

### The Bacterial Communities in Cold Tap and Shower Water Were Significantly More Diverse Than That in Hot Tap Water

**FIGURE 4:**
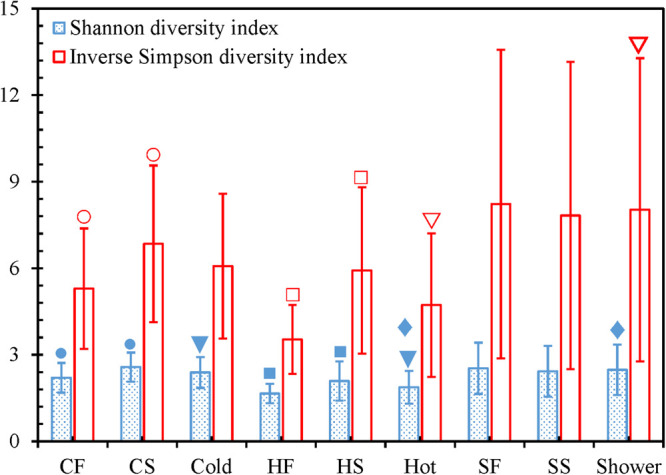
Shannon and Inverse Simpson diversity indices. **CF**: Cold tap water-First draw. **CS**: Cold tap water-Second draw. **Cold**: Cold tap water. **HF**: Hot tap water-First draw. **HS**: Hot tap water-Second draw. **Hot**: Hot tap water. **SF**: Shower water-First draw. **SS**: Shower water-Second draw. **Shower**: Shower water. Column heights: Arithmetic means. Error bars: Standard deviations (*n* = 16 for **CF**, **CS**, **HF**, **HS**, **SF**, and **SS**. *n* = 32 for **Cold**, **Hot**, and **Shower**). We used Mothur to calculate the indices for each water sample and SPSS^®^ to determine whether the differences of the indices for each of the following pairs were statistically significant: **CF** versus **CS**, **HF** versus **HS**, **SF** versus **SS**, **Cold** versus **Hot**, **Cold** versus **Shower**, and **Hot** versus **Shower**. A pair of indices marked with the same symbol are significantly different (*p* < 0.05).

For both cold and hot tap water, the second draws had significantly greater Shannon and Inverse Simpson diversity indices than the first draws (two-tailed paired *t*-test with SPSS^®^, *p* < 0.05) (Figure 4 and Supplementary Figures 2 and 3). Therefore, the second draws for tap water had a greater bacterial community diversity (richness and evenness) and represented different bacterial niches from the first draws (Serrano-Suárez et al., 2013; Waak et al., 2019). Indeed, we identified more OTUs from the second draws of tap water than from the first draws (i.e., the second draws had a greater richness) (Figure 1 and Supplementary Table 11) (Liao et al., 2013). In addition, the rarefaction curves for the second draws of tap water had greater slopes than the first draws (Supplementary Table 5 and Supplementary Figure 1), confirming that the second draws of tap water had a greater evenness. The greater diversity of the bacterial communities in the second draws of tap water might be due to the greater water temperatures of the second draws. The mean temperatures for the second draws of cold and hot tap water were 30.1 ± 3.3°C and 46.9 ± 1.2°C, respectively. The mean temperatures for the first draws of cold (20.9 ± 1.1°C) and hot (30.4 ± 5.3°C) tap water were much lower. For both cold and hot tap water, the first draws were stagnant water, while the second draws were representative water. Therefore, in this HPS simulator, stagnant tap water had a lower bacterial community diversity than the representative water because of the water temperature difference. The greater diversity of the bacterial community in the second draws of tap water implies that a greater water temperature might promote the (re)growth and colonization of opportunistic pathogens in building PPSs. On the other hand, the first and second draws for shower water had comparable Shannon and Inverse Simpson diversity indices (two-tailed paired *t*-test with SPSS^®^, *p* > 0.05) (Figure 4 and Supplementary Figures 2 and 3), numbers of OTUs (Figure 1 and Supplementary Table 11), and rarefaction curve slopes (Supplementary Table 5 and Supplementary Figure 1). As a result, the two draws for shower water had similar bacterial community diversities because those two draws were collected consecutively and had comparable temperatures (38.3 ± 2.5°C and 38.5 ± 3.1°C for the first and second draws, respectively).

The Shannon diversity indices of cold tap (2.39 ± 0.54) and shower (2.48 ± 0.87) water were significantly greater than that of hot tap water (1.87 ± 0.57) (SPSS^®^, *p* for the Kruskal–Wallis test < 0.001, *p* for the *post hoc* test with Bonferroni correction < 0.01) (Figure 4). The Inverse Simpson diversity index for shower water (8.03 ± 5.26) was also significantly greater than that of hot tap water (4.73 ± 2.49) (SPSS^®^, *p* for the Kruskal–Wallis test 0.020, *p* for the *post hoc* test with Bonferroni correction 0.041). In addition, cold tap water (6.07 ± 2.51) had a greater Inverse Simpson diversity index than hot tap water even though the difference was not statistically significant (SPSS^®^, *p* for the Kruskal–Wallis test 0.020, *p* for the *post hoc* test with Bonferroni correction 0.281). Furthermore, cold tap and shower water had more OTUs (Figure 1 and Supplementary Table 11) and greater rarefaction curve slopes (Supplementary Table 5 and Supplementary Figure 1) than hot tap water. Therefore, the bacterial communities in cold tap and shower water were more diverse than that in hot tap water. Similarly, in a research center in Germany, cold tap water had significantly greater Shannon (2.43 ± 0.16) and Inverse Simpson (8.88 ± 2.09) diversity indices than hot drinking water from a showerhead (Shannon 1.56 ± 0.14; Inverse Simpson 3.82 ± 0.53) (Lesnik et al., 2016). In the same research center, cold tap water had a significantly greater bacterial community richness (mean rank in a rank abundance plot 55, a steeper slope) than hot drinking water (mean rank 40, a flatter slope) (Henne et al., 2013). On the other hand, the comparable Shannon and Inverse Simpson diversity indices (SPSS^®^, *p* for the Kruskal–Wallis test ≤ 0.020, *p* for the *post hoc* test with Bonferroni correction 1.000) (Figure 4), numbers of OTUs (Figure 1 and Supplementary Table 11), and rarefaction curve slopes (Supplementary Table 5 and Supplementary Figure 1) for cold tap and shower water indicate that they had similar bacterial community diversities.

### Water Temperature, Total Chlorine Residual Concentration, and the Age of the HPS Simulator Dictated Bacterial Community Diversity

We used four independent variables (absolute sampling time or the age of the simulator, water temperature, total chlorine residual concentration, and ambient temperature) to predict the Shannon and Inverse Simpson diversity indices (dependent variables) using multiple linear regression (Table 1). The four independent variables together significantly predict the dependent variables (*R*^2^ > 0.400, *p* < 0.001). The absolute sampling time (i.e., the age of the simulator) had positive unstandardized coefficients and statistically significant correlations with both diversity indices (*p* < 0.001). Therefore, the diversity of bacterial community increased with the age of the simulator. When the simulator aged, pipe biofilms and loose deposits might have developed in microbial and physical complexity, releasing bacteria communities with a greater diversity to the corresponding bulk water (Rittmann and McCarty, 2001; Martiny et al., 2003; Henne et al., 2012; Liu et al., 2014, 2018; Petrova and Sauer, 2016; Prest et al., 2016a; Potgieter et al., 2018; Chan et al., 2019). The unstandardized coefficients for total chlorine residual concentration were negative for both diversity indices. A previous study similarly found that both bulk drinking water and biofilms in a chloraminated DWDS in the US had significantly lower Shannon and Inverse Simpson diversity indices than those in a Norwegian DWDS without a disinfectant residual (*p* ≤ 0.0001) (Waak et al., 2019). In another study, total bacterial numbers (16S rRNA gene copy numbers) in the effluents of simulated household water heaters were negatively correlated with disinfectant (chlorine and chloramine) residual concentrations in upstream simulated DWDSs (Spearman’s rank correlation coefficients between −0.752 and −0.458, *p* < 0.001) (Wang et al., 2015). Therefore, a greater disinfectant residual concentration effectively suppressed the diversity of bacterial community. Water temperature had a significant positive correlation with the Inverse Simpson diversity index (*p* 0.011) but did not have a significant linear correlation with the Shannon diversity index (*p* 0.377). The Inverse Simpson diversity index is more appropriate when dominant species are more important in a microbial community, while the Shannon diversity index is more appropriate when rare and dominant species are equally important (Morris et al., 2014). In the current study, a few dominant groups of bacteria represented the majority of the whole community (Figures 2 and 3, Supplementary Tables 8 and 9, and Supplementary Figures 7 to 10), suggesting that the dominant species were more important. In addition, the Inverse Simpson diversity index is superior to the Shannon diversity index because the former considers the difference in sampling efforts (Pielou, 1975). Therefore, the diversity of bacterial communities significantly increased with water temperature (revealed by the Inverse Simpson diversity index). A previous study similarly found that the richness (observed OTUs) of drinking water bacterial community had a statistically significant, positive correlation with water temperature (Pearson’s *R* 0.74, *p* < 0.05) (Pinto et al., 2014).

**TABLE 1:** A multiple linear regression model predicting the Shannon and Inverse Simpson diversity indices of the water samples with four independent variables (absolute sampling time, water temperature, total chlorine residual concentration, and ambient temperature).

Regression result	Shannon diversity index	Inverse Simpson diversity index
Coefficient of determination (*R*^2^)	0.414	0.434
Adjusted *R*^2^	0.389	0.409
*F*-ratio (*p*) for the overall model	16.095 (< 0.001*)	17.412 (< 0.001*)
Unstandardized coefficient (*p*) of absolute sampling time	0.002 (< 0.001*)	0.014 (< 0.001*)
Unstandardized coefficient (*p*) of water temperature	0.006 (0.377)	0.091 (0.011*)
Unstandardized coefficient (*p*) of total chlorine residual concentration	−1.531 (0.005*)	−3.516 (0.210)
Unstandardized coefficient (*p*) of ambient temperature	−0.003 (0.667)	−0.056 (0.103)
Unstandardized coefficient (*p*) of the constant	1.679 (< 0.001*)	1.215 (0.423)

Analyzed with SPSS^®^. *Statistical significance (*p* < 0.05).

This study found that the diversity of bacterial communities significantly increased with water temperature (revealed by the Inverse Simpson diversity index) (Table 1) but the bacterial community in hot tap water was less diverse than those in cold tap and shower water (Figures 1 and 4, Supplementary Tables 5 and 11, and Supplementary Figures 1 to 3). The paradoxical results might be due to three reasons. First, other environmental factors such as the formation of pipe biofilms/deposits than water temperature might make the bacterial community in hot tap water less diverse. In addition, only a small portion of drinking water bacteria could adapt to the relatively high temperature of hot tap water (Supplementary Table 1), thus decreasing the bacterial community diversity of hot tap water. Second, the Inverse Simpson diversity index of the bacterial communities for all water types (hot tap, cold tap, and shower water) increased with water temperature (Supplementary Figure 11). However, the Inverse Simpson diversity indices of many hot tap water samples (particularly the first draw samples) were lower than those of cold tap and shower water samples. Indeed, the arithmetic mean of the Inverse Simpson diversity indices for hot tap water (4.73) was lower than those for cold tap (6.07) and shower (8.03) water (Figure 4). Since the Inverse Simpson diversity index for all water types increased with water temperature (Supplementary Figure 11), the multiple linear regression reveals that for the “pooled” water samples (e.g., mixed datapoints for all 96 water samples), the Inverse Simpson diversity index significantly increased with water temperature (Table 1) although the Inverse Simpson diversity index of hot tap water was lower than those of cold tap and shower water. Third, water temperatures of some first draw samples of hot tap water was comparable to or even lower than those of some shower water samples and some second draw samples of cold tap water (Supplementary Table 1 and Supplementary Figure 3B). Therefore, water temperature of a hot tap water sample was not necessarily greater than that of a cold tap or shower water sample.

We used the Shannon and Inverse Simpson diversity indices of cold and hot tap water to predict those of shower water with multiple linear regression (Supplementary Table 12). This regression significantly predicts the diversity indices of shower water (SPSS^®^, *R*^2^ 0.451 and 0.333 for the Shannon and Inverse Simpson diversity indices, respectively; *p* < 0.005). However, this regression explains only less than 50% of the variation in the diversity indices of shower water, and the *p*-values for the correlation coefficients for the diversity indices of hot tap water are greater than 0.05. In addition, the relative abundance of dominant bacterial groups in cold and hot tap water seemed unrelated to those in shower water (Figures 2 and 3 and Supplementary Figures 7A and 9). Furthermore, only approximately 26% of the OTUs identified from cold tap and shower water were shared by those two types of water, while for hot tap and shower water, this figure was even lower (approximately 22%) (Figure 1). Therefore, the bacterial community in shower water was more than a simple combination of those in cold and hot tap water although shower water was simply a blend of cold and hot tap water. As discussed above, two underlying mechanisms could make the bacterial community in shower water more than a simple mixture of those in cold and hot tap water. First, the biofilms and loose deposits adapted to the specific local environment of the showerhead might make the bacterial community in shower water different from those in cold and hot tap water (Bollin et al., 1985; Feazel et al., 2009; Lu et al., 2017). Second, the sudden changes in critical physicochemical water quality parameters, especially water temperature, when cold and hot tap water was mixed in the showerhead might also make the bacterial community in shower water unique (Henne et al., 2013). Other reasons might count as well. For instance, the hydraulic properties of Faucet 4 and the showerhead in the current study were significantly different. The unique hydraulic structure of the showerhead could potentially contribute to the unique bacterial community in shower water.

### The Bacterial Community Structures of Cold Tap and Shower Water Were Significantly Different From That of Hot Tap Water

**FIGURE 5:**
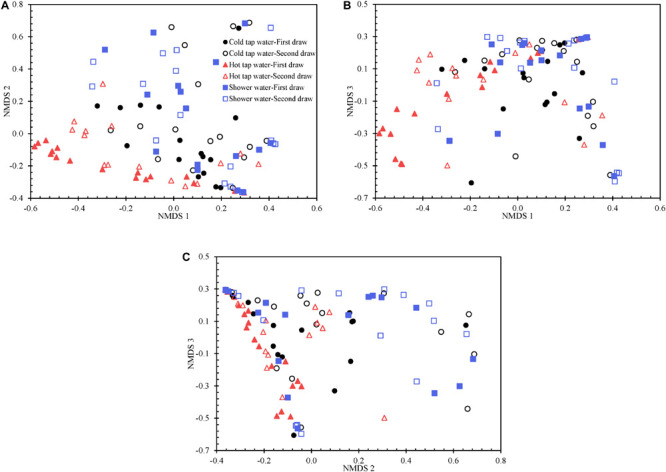
The 3D NMDS of the Yue and Clayton theta distance matrix for all water samples visualized using three two-dimensional plots. **(A)** NMDS 1 versus NMDS 2. **(B)** NMDS 1 versus NMDS 3. **(C)** NMDS 2 versus NMDS 3.

We visualized the Yue and Clayton theta distance matrix with a 3D PCoA (Supplementary Figure 12) and a 3D NMDS (Figure 5 and Supplementary Figure 13). The NMDS (*R*^2^ 0.914) is better than the PCoA (*R*^2^ 0.833) in explaining the distance matrix. Indeed, the lowest stress value for the NMDS is 0.123, suggesting that the NMDS well represents the distance matrix in the reduced dimensions (Clarke, 1993). Any groups of water samples (i.e., the first draw of cold tap water, second draw of cold tap water, first draw of hot tap water, second draw of hot tap water, first draw of shower water, and second draw of shower water) diverge from one another in the PCoA and NMDS ordination plots. However, the bacterial communities for any of the following groups were homogenous or had similar variations: the first draw versus the second draw of cold tap water, the first draw versus the second draw of hot tap water, the first draw versus the second draw of shower water, and cold tap water versus hot tap water versus shower water (HOMOVA test, *p* > 0.05) (Supplementary Table 13). Therefore, the bacterial communities in the first and second draws for any water type were equally stable. In addition, the bacterial communities from the different endpoints of the HPS simulator (i.e., cold tap, hot tap, and showerhead) had comparable stabilities (HOMOVA test, *p* 0.072). Furthermore, we applied the HOMOVA test to determine whether the variations in bacterial communities in the water samples of March to July in 2012 (i.e., the “early” samples) and those of March to July in 2013 (i.e., the “late” samples) were homogeneous. The bacterial community in the early cold tap water samples were more stable than that in the late cold tap water samples (HOMOVA test, *p* 0.012) (Supplementary Table 13). However, the bacterial communities in the early and late samples of either hot tap or shower water were equally stable or had similar variations (HOMOVA test, *p* > 0.05). We hypothesize that the bacterial community in cold tap water became less stable when the HPS simulator aged and the pipe biofilms and loose deposits matured. On the other hand, since hot tap and shower water contained heated cold tap water, the stabilities of the bacterial communities of hot tap and shower water responded minimally to the age of the simulator. Presumably, the effect of the heating of cold tap water masked the effect of the age of the simulator on bacterial community stabilities of hot tap and shower water.

**FIGURE 6:**
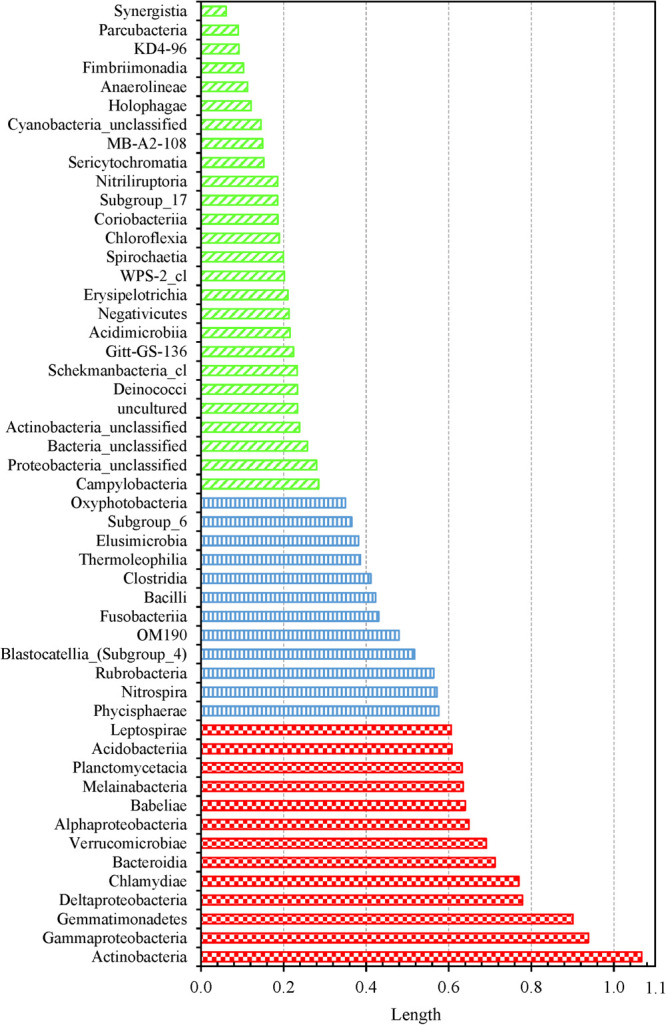
Shifting of water samples in the 3D NMDS space by bacterial classes. Fifty one classes were identified from all water samples. Classes with lengths greater than 0.6, between 0.3 and 0.6, and less than 0.3: red large checker board, blue vertical stripes, and green wide upward diagonal stripes, respectively.

We determined which OTUs and classes of bacteria were more responsible for the specific distribution of the water samples in the NMDS ordination plot (Figure 5 and Supplementary Figure 13). OTUs 0001, 0009, 0029, 0002, 0024, 0026, 0027, 0033, 0031, and 0017 (descending order of significance) were the top ten most important OTUs that moved the samples in the NMDS space (Supplementary Table 14). Actinobacteria, Gammaproteobacteria, Gemmatimonadetes, Deltaproteobacteria, Chlamydiae, and Bacteroidia (descending order of significance) were the top five most important classes that shifted the bacterial communities in the NMDS plot (Supplementary Table 15 and Figure 6). In addition, the absolute sampling time or the age of the HPS simulator (length 0.913) is more significant than total chlorine residual concentration (length 0.471) and water temperature (length 0.186) in shifting the bacterial communities in the NMDS space (Table 2). Specifically, the *p*-value for the correlation coefficient between water temperature and any of the three NMDS axes is greater than 0.05, suggesting that water temperature was not an important parameter shifting the bacterial communities in the NMDS space.

**TABLE 2:** Shifting of bacterial communities in the 3D NMDS space by water temperature, absolute sampling time, and total chlorine residual concentration.

Factor	Spearman’s rank correlation coefficient			Length
	NMDS 1 (*p*)	NMDS 2 (*p*)	NMDS 3 (*p*)	
Water temperature (°C)	−0.056 (0.585)	0.081 (0.427)	0.158 (0.124)	0.186
Absolute sampling time (d)	−0.567 (< 0.001*)	0.678 (< 0.001*)	−0.228 (0.026*)	0.913
Total chlorine residual (mg Cl_2_⋅L^–1^)	0.364 (< 0.001*)	−0.289 (0.004*)	0.0785 (0.444)	0.471

Analyzed with Mothur. *Statistical significance (*p* < 0.05). NMDS 1, NMDS 2, and NMDS 3: Axes 1, 2, and 3, respectively, of the NMDS space. Length = [(Coefficient for NMDS 1)^2^ + (Coefficient for NMDS 2)^2^ + (Coefficient for NMDS 2)^2^]^0.5^.

The first and second draws of either cold tap or shower water occupy approximately the same spaces in either the PCoA (Supplementary Figure 12) or the NMDS (Figure 5 and Supplementary Figure 13) ordination plot (even though they do diverge). However, for hot tap water, the first and second draws occupy apparently different spaces. Indeed, the two draws of hot tap water have significantly different centroids in either ordination plot (AMOVA test, *p* 0.016), but the spatial separation of the first and second draws for either cold tap or shower water is minimal (AMOVA test, *p* > 0.05) (Supplementary Table 13). The temperature difference between the two draws of either cold tap or shower water is smaller than that between the two draws of hot tap water (Supplementary Table 1). Those temperature differences might explain why the two draws of hot tap water have different centroids in the ordination plots while the two draws of either cold tap or shower water occupy approximately the same spaces.

Cold tap and shower water approximately cluster and are spatially separate from hot tap water in either ordination plot (Figure 5 and Supplementary Figures 12 and 13). The AMOVA test confirms that the bacterial community structure of hot tap water diverged significantly from those of cold tap and shower water (*p* < 0.001), while the bacterial community structures for cold tap and shower water were comparable (*p* 0.373) (Supplementary Table 13). Similarly, a study revealed remarkably different bacterial community structures for cold and hot drinking water from a laboratory using DNA fingerprinting (Henne et al., 2013). Another study found distinct microbial community structures between cold and hot tap water systems in an office building using Illumina MiSeq (Inkinen et al., 2016). With a culture-based method, a study found that only 2% of the acridine orange direct counts in cold tap water (supply of hot tap water) from an apartment building were culturable heterotrophic bacteria, but the figure for hot tap water from the same building was much greater (38%) (Bagh et al., 2004). Even though the bacterial community of hot tap water in the current study was spatially away from those of cold tap and shower water in the two ordination plots (Figure 5, Supplementary Figures 12 and 13, and Supplementary Table 13), the best enterotype number based on the DMM model is one, indicating that the bacteria for all water samples belonged to only one metacommunity. On the other hand, the AMOVA test indicates that the community structures of the early samples (March to July in 2012) were significantly different from those of the late samples (March to July in 2013) for cold tap, hot tap, and shower water (*p* < 0.001) (Supplementary Table 13). Therefore, the age of the HPS simulator significantly affected the bacterial community structures.

**FIGURE 7:**
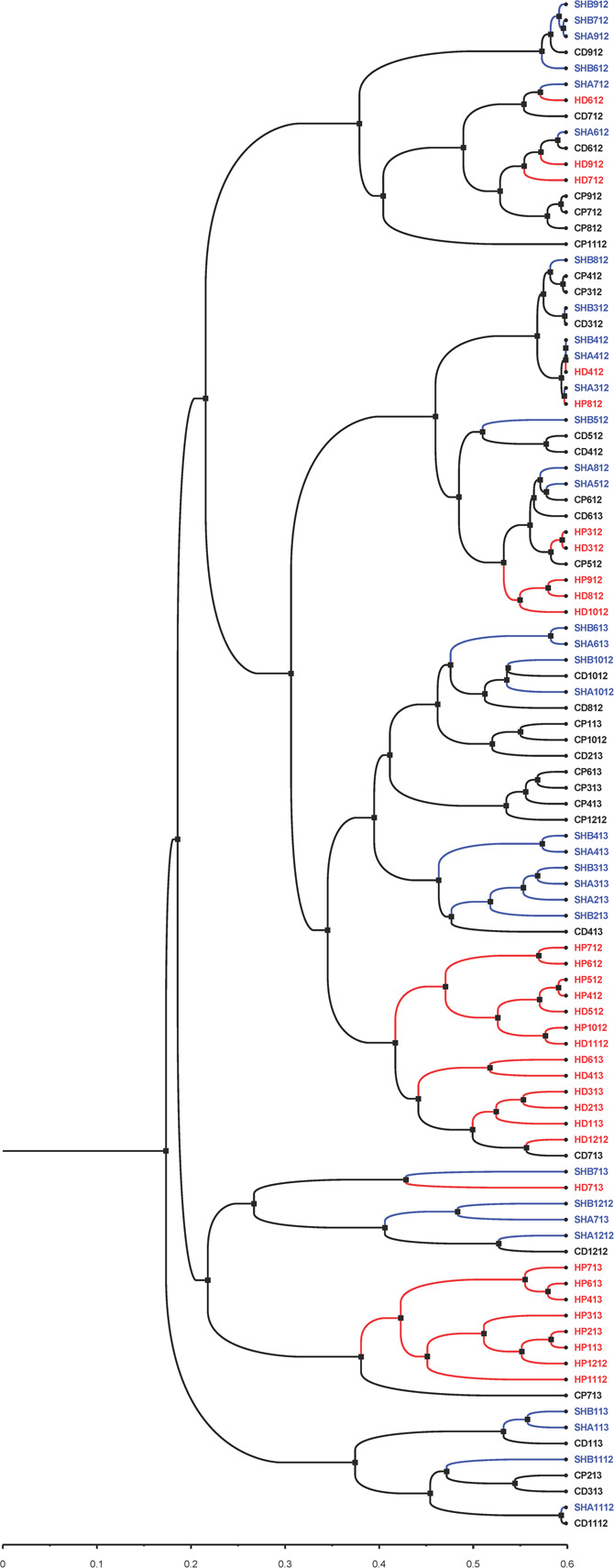
A phylogenetic dendrogram for cold tap, hot tap, and shower water. Visualized with FigTree (version 1.4.4). Letters in the sample IDs: **CP**: Cold tap water-First draw. **CD**: Cold tap water-Second draw. **HP**: Hot tap water-First draw. **HD**: Hot tap water-Second draw. **SHA**: Shower water-First draw. **SHB**: Shower water-Second draw. Numbers in the sample IDs: Sampling dates in the format of “MYY” or “MMYY,” where the “M” or “MM” stands for month and the “YY” stands for the year (2012 and 2013). For instance, the sample ID of “SHB1112” stands for the second draw of shower water sampled in November 2012.

In addition to the PCoA and NMDS ordination plots (Figure 5 and Supplementary Figures 12 and 13), we generated a phylogenetic dendrogram from the Yue and Clayton theta distance matrix to visualize the similarity among the bacterial community structures of groups of samples (Figure 7). The first and second draws of either cold tap or shower water cluster in the dendrogram. The parsimony, unweighted UniFrac, and weighted UniFrac tests all indicate that the bacterial community structures of the first and second draws of either cold tap or shower water were similar (*p* > 0.05) (Table 3). When ignoring the branch length of the dendrogram (i.e., the parsimony test), the bacterial community structures between the first and second draws of hot tap water were similar (*p* 0.085). However, when incorporating the branch length (i.e., the two UniFrac tests), the difference in the bacterial community structures between the two draws of hot tap water was significant (*p* < 0.01). Since the branch length is important in determining the clustering of samples in a phylogenetic dendrogram, the first and second draws of hot tap water had distinct bacterial community structures.

**TABLE 3:** Parsimony and UniFrac tests on the clustering of water samples in the phylogenetic dendrogram.

Comparison of groups	Parsimony		Unweighted UniFrac		Weighted UniFrac	
	Score	*p*	Score	*p*	Score	*p*
Cold tap water: First draw versus second draw	9	0.461	0.705	0.227	0.419	0.527
Hot tap water: First draw versus second draw	7	0.085	0.752	0.002*	0.589	0.009*
Shower water: First draw versus second draw	14	0.999	0.499	0.932	0.231	0.991
Cold tap water versus hot tap water	10	< 0.001*	0.802	< 0.001*	0.536	< 0.001*
Cold tap water versus shower water	20	0.596	0.638	0.128	0.328	0.553
Hot tap water versus shower water	10	< 0.001*	0.820	< 0.001*	0.570	< 0.001*

Analyzed with Mothur. *Statistical significance (*p* < 0.05).

Hot tap water samples cluster with themselves in the dendrogram, while the samples of cold tap water cluster with those of shower water (Figure 7). The parsimony, unweighted UniFrac, and weighted UniFrac tests all indicate that the bacterial community in hot tap water had a significantly different structure from those in cold tap and shower water (*p* < 0.001) but the bacterial community structures of cold tap and shower water were similar (*p* > 0.05) (Table 3).

### Limitations and Future Research

The major limitation of this work is the lack of comparing bacterial communities in multiple PPSs to generalize the results. Restricted by privacy, management policy, and research resources, this study monitored the dynamics of bacterial communities in tap and shower water in only one location (i.e., a single HPS simulator). Future studies should include multiple PPSs in different geographical locations to reach more generalized conclusions and discover the universal characteristics of the dynamics of bacterial communities in drinking water of building PPSs.

This work revealed that the fluctuations of dominant phyla and classes in the water samples had limited seasonal variations (Supplementary Figures 7B to 7G and 8). In addition, no significant linear correlations were found between ambient temperature and both Shannon and Inverse Simpson diversity indices of the water samples (*p* > 0.05) (Table 1). By contrast, previous studies found clear seasonal variations in bacterial communities of cold drinking water (McCoy and VanBriesen, 2012; Henne et al., 2013; Pinto et al., 2014; Prest et al., 2016b; Vavourakis et al., 2020). We hypothesize that the lack of clear seasonal variations in bacterial communities in the current study could be because the HPS simulator delivered temperature-stable tap and shower water to the bathroom. Indeed, the temperature of cold tap water (both first and second draws) was insensitive to the changes in ambient temperature (Supplementary Figure 4). In addition, the temperature of either the first or the second draw of cold tap water had limited seasonal variations (Supplementary Table 1). The temperatures of hot tap and shower water were also relatively stable over the 16-month sampling period. Temperature is the most important abiotic factor governing the structure of a microbial community. Therefore, the bacterial communities in the simulator lacked significant seasonal variations. Future studies need to confirm whether water temperature was the major factor that contributed to the lack of seasonal variations of the bacterial communities. The temporal dynamics of the drinking water microbial communities in the HPS simulator should also be further explored.

This work measured or recorded only four environmental parameters that could affect the diversity of the bacterial communities in the HPS simulator, while other parameters could also affect the diversity. Indeed, the four environmental parameters in the multiple linear regression model explain only approximately 40% of the variation in the Shannon and Inverse Simpson diversity indices (Table 1). Other physicochemical and biological environmental parameters such as pH, conductivity, organic carbon concentration, nutrient (nitrogen and phosphorus) concentration, and biofilm formation might explain the remaining variation (Hwang et al., 2012; Pinto et al., 2012, 2014; Henne et al., 2013; Inkinen et al., 2016). Future studies need to explore how other environmental parameters affect the diversity of the bacterial communities.

## Conclusion

We sampled cold tap, hot tap, and shower water monthly from a HPS simulator in a bathroom for 16 months and monitored the bacterial community structures in those samples using 16S-rRNA-gene-based high-throughput DNA sequencing. We identified 24 phyla and 248 families from the 96 water samples. At the phylum level, Proteobacteria, Actinobacteria, and Bacteroidetes were dominant. The top five most abundant families were Burkholderiaceae, Sphingomonadaceae, unclassified Alphaproteobacteria, unclassified Corynebacteriales, and Mycobacteriaceae. The Shannon and Inverse Simpson diversity indices of the water samples increased with water temperature and the age of the simulator but decreased with total chlorine residual concentration. Ambient temperature did not have a significant linear correlation with the diversity indices. In addition, the relative abundance of dominant phyla and classes in cold tap, hot tap, and shower water all significantly fluctuated over the 16-month sampling period, but the fluctuations lacked a clear seasonal trend. Therefore, the bacterial communities in the simulator had limited seasonal variations. Hot tap water had a significantly lower bacterial community diversity than cold tap and shower water. Moreover, the bacterial community structure of hot tap water was significantly different from those of cold tap and shower water, while cold tap and shower water had similar bacterial community structures. The bacterial community compositions for the first and second draws of shower water were comparable, but the two draws of either cold or hot tap water had distinct community compositions. In conclusion, the bacterial community in hot tap water was less diverse than and had a distinct structure from those in cold tap and shower water. Therefore, one needs to simultaneously monitor the dynamics of the microbial communities in cold tap, hot tap, and shower water to comprehensively understand microbial drinking water quality in a PPS.

## Data Availability Statement

The datasets presented in this study can be found in an online repository. The name of the repository and accession numbers can be found in the article.

## Author Contributions

CZ and KQ performed data analysis and contributed equally to this article. CZ drafted and revised the article. IS and HB conducted the experiments and collected the data. DL constructed the home plumbing system simulator and provided the parameters of this simulator. HB, JSD, and DL participated in revision and polishing. JL was the principal investigator who designed the study and finalized the article. All authors contributed to the article and approved the submitted version.

## Conflict of Interest

CZ was employed by Pegasus Technical Services, Inc., (Cincinnati, Ohio, United States). The remaining authors declare that the research was conducted in the absence of any commercial or financial relationships that could be construed as a potential conflict of interest.
