# Supplementary material for: The Bacterial Community Diversity of Bathroom Hot Tap Water Was Significantly Lower Than That of Cold Tap and Shower Water
Source: Front Microbiol. 2021 Apr 23;12:625324. doi: 10.3389/fmicb.2021.625324 (PMC8102780; doi:10.3389/fmicb.2021.625324)
Supplement: Supplementary file 1 [file Presentation_1.zip › Supplementary material for the Proof/Figure S10 Krona.html]

Javascript must be enabled to view this page.

magnitude

All 96 samples

 2104926

 2104926

 652549

 652460

 651683

 644759

 644683

 9

 12

 10

 5

 9

 31

 3848

 3776

 72

 1479

 603

 424

 351

 51

 26

 14

 6

 4

 261

 207

 18

 36

 182

 182

 157

 157

 154

 154

 158

 103

 55

 97

 81

 12

 4

 76

 76

 34

 34

 37

 31

 6

 30

 30

 18

 18

 14

 14

 13

 13

 11

 11

 11

 11

 11

 11

 10

 10

 15

 15

 24

 8

 16

 8

 8

 7

 7

 228

 228

 6

 6

 21

 21

 3

 3

 7

 3

 2

 2

 4

 4

 267

 238

 238

 29

 18

 7

 2

 1

 1

 391

 232

 99

 73

 28

 15

 6

 2

 6

 1

 1

 1

 98

 76

 20

 1

 1

 12

 12

 12

 12

 10

 10

 15

 8

 7

 8

 5

 2

 1

 4

 4

 68

 68

 65

 2

 1

 14

 9

 8

 1

 2

 2

 1

 1

 1

 1

 1

 1

 10

 8

 8

 2

 2

 8

 8

 7

 1

 5

 5

 3

 2

 3

 3

 3

 3

 3

 3

 8

 8

 8

 14

 14

 14

 14

 47

 29

 14

 12

 2

 13

 13

 2

 2

 18

 11

 11

 7

 7

 16

 16

 7

 7

 5

 5

 3

 3

 1

 1

 5

 5

 5

 5

 4

 4

 4

 4

 2

 2

 2

 2

 1

 1

 1

 1

 1249347

 569705

 498943

 447410

 258665

 147867

 12326

 23417

 2570

 584

 435

 291

 188

 182

 141

 106

 104

 96

 74

 71

 48

 30

 26

 24

 33

 17

 16

 25

 8

 7

 8

 5

 5

 5

 4

 6

 9

 3

 2

 1

 1

 1

 1

 1

 2

 1

 1

 2

 1

 28683

 28683

 10682

 8128

 1375

 817

 177

 71

 37

 32

 34

 5

 2

 4

 6569

 6301

 234

 9

 4

 4

 10

 7

 4886

 4884

 1

 1

 283

 239

 29

 8

 7

 61

 61

 184

 184

 43

 33

 10

 30

 30

 30

 30

 16

 16

 15

 15

 12

 12

 6

 6

 6

 6

 6

 6

 5

 5

 5

 5

 4

 4

 2

 2

 3

 3

 2

 2

 54464

 46323

 45941

 287

 43

 41

 5

 6

 8139

 4493

 3051

 280

 254

 20

 29

 8

 4

 2

 2

 6186

 6186

 6186

 3799

 3799

 3799

 1143

 1143

 1027

 85

 15

 16

 972

 972

 971

 1

 1869

 1827

 913

 349

 340

 61

 69

 34

 23

 19

 8

 3

 2

 5

 1

 38

 18

 7

 5

 3

 2

 2

 1

 3

 3

 1

 1

 1812

 1812

 1707

 78

 19

 5

 3

 142

 142

 142

 145

 144

 144

 1

 1

 88

 81

 81

 5

 5

 2

 2

 34

 34

 34

 31

 31

 20

 11

 17

 17

 16

 1

 14

 14

 14

 13

 13

 7

 3

 2

 1

 5

 5

 5

 7

 5

 5

 2

 1

 1

 4

 4

 4

 3

 3

 3

 3

 3

 3

 6

 6

 6

 2

 2

 2

 2

 2

 2

 1

 1

 1

 673026

 247826

 168071

 166464

 486

 432

 378

 102

 36

 32

 28

 22

 13

 11

 10

 8

 7

 7

 9

 5

 5

 3

 5

 1

 7

 56523

 56046

 309

 92

 45

 9

 7

 7

 4

 3

 1

 16701

 16701

 3750

 3303

 403

 27

 4

 13

 2204

 2204

 289

 169

 45

 42

 27

 5

 1

 123

 123

 27

 26

 1

 35

 23

 12

 13

 13

 12

 12

 10

 10

 7

 7

 7

 7

 11

 7

 3

 1

 15

 15

 12

 12

 6

 6

 5

 5

 4

 4

 1

 1

 267619

 267619

 92158

 52068

 38528

 32559

 24348

 14929

 8811

 1935

 423

 317

 215

 204

 126

 77

 59

 50

 49

 44

 56

 39

 51

 32

 31

 26

 42

 21

 20

 19

 24

 16

 30

 15

 28

 13

 13

 12

 11

 10

 20

 10

 10

 24

 7

 18

 6

 12

 6

 10

 5

 28

 5

 4

 4

 11

 12

 3

 14

 1

 68703

 68703

 68695

 5

 2

 1

 41525

 41525

 41525

 10488

 10488

 10488

 13129

 11750

 8632

 2483

 481

 70

 22

 35

 11

 8

 5

 3

 1367

 1367

 12

 4

 6

 2

 8120

 8120

 8112

 3

 2

 2

 1

 2934

 2934

 2934

 4905

 4905

 4904

 1

 1917

 1554

 1554

 319

 319

 40

 39

 1

 4

 4

 1215

 1215

 1215

 2482

 2482

 1142

 979

 314

 25

 10

 4

 3

 3

 2

 766

 766

 766

 475

 472

 472

 2

 2

 1

 1

 130

 130

 129

 1

 81

 81

 81

 63

 63

 63

 78

 78

 66

 11

 1

 26

 26

 26

 294

 294

 294

 21

 21

 21

 19

 19

 19

 19

 19

 19

 20

 20

 20

 13

 13

 13

 18

 15

 15

 3

 3

 12

 12

 12

 11

 11

 11

 10

 10

 10

 9

 9

 9

 39

 39

 39

 7

 7

 7

 5

 5

 5

 5

 5

 5

 10

 10

 10

 12

 12

 12

 4

 4

 4

 3

 3

 3

 3

 3

 3

 3

 3

 3

 3

 3

 3

 1

 1

 1

 1

 1

 1

 1

 1

 1

 1

 1

 1

 5872

 5834

 4710

 4710

 1124

 1103

 21

 9

 9

 9

 8

 8

 8

 6

 6

 6

 5

 5

 5

 5

 3

 3

 2

 2

 3

 2

 2

 1

 1

 2

 2

 2

 729

 729

 729

 729

 15

 15

 15

 15

 111224

 111224

 77146

 77142

 48309

 24047

 2304

 2209

 36

 42

 16

 13

 83

 11

 6

 9

 5

 10

 7

 4

 7

 5

 8

 6

 3

 1

 1

 4

 4

 33423

 33403

 32515

 728

 78

 50

 28

 3

 1

 17

 14

 3

 3

 3

 393

 350

 151

 128

 36

 34

 1

 33

 18

 14

 1

 5

 5

 3

 3

 2

 2

 106

 37

 37

 65

 26

 22

 11

 3

 3

 3

 3

 1

 1

 155

 50

 37

 5

 8

 67

 67

 24

 24

 10

 10

 2

 2

 2

 1

 1

 1

 1

 1

 69745

 67243

 67061

 67061

 67061

 75

 75

 75

 38

 38

 38

 29

 29

 29

 28

 28

 28

 5

 5

 5

 7

 7

 7

 1934

 1934

 1934

 1934

 374

 264

 264

 264

 110

 106

 101

 5

 4

 4

 174

 174

 174

 174

 19

 19

 19

 19

 1

 1

 1

 1

 6858

 6858

 6858

 6858

 6551

 272

 21

 12

 2

 4286

 3418

 2497

 1330

 1329

 1

 270

 266

 4

 158

 158

 136

 136

 141

 141

 265

 52

 66

 51

 67

 14

 9

 6

 41

 41

 51

 51

 21

 21

 47

 16

 13

 5

 5

 3

 2

 3

 10

 10

 9

 9

 9

 9

 7

 7

 1

 1

 1

 1

 908

 547

 469

 78

 224

 188

 13

 12

 6

 5

 77

 69

 6

 2

 26

 15

 11

 33

 33

 1

 1

 13

 13

 13

 846

 843

 321

 245

 35

 28

 13

 102

 87

 10

 3

 1

 1

 75

 66

 3

 2

 2

 1

 1

 157

 47

 42

 28

 23

 8

 5

 3

 1

 63

 41

 13

 4

 3

 2

 70

 70

 47

 37

 3

 4

 2

 1

 5

 5

 3

 3

 3

 3

 3

 20

 20

 20

 19

 1

 2

 2

 2

 1

 1

 2185

 2185

 2185

 2185

 2185

 1276

 1276

 1276

 1128

 1128

 140

 138

 2

 8

 8

 900

 895

 895

 895

 895

 5

 5

 5

 5

 1370

 1370

 1310

 834

 831

 1

 2

 476

 476

 25

 25

 22

 3

 12

 10

 9

 1

 2

 2

 23

 7

 6

 1

 13

 13

 3

 3

 2247

 2073

 1767

 1767

 893

 698

 116

 46

 14

 99

 77

 77

 11

 11

 6

 6

 2

 2

 3

 3

 169

 169

 59

 110

 26

 26

 24

 1

 1

 12

 12

 12

 161

 161

 161

 161

 13

 12

 12

 12

 1

 1

 1

 673

 673

 673

 551

 551

 63

 63

 41

 41

 9

 9

 7

 7

 2

 2

 676

 502

 492

 492

 455

 31

 6

 5

 5

 5

 5

 5

 5

 130

 104

 104

 88

 8

 6

 2

 26

 26

 26

 42

 41

 41

 41

 1

 1

 1

 1

 1

 1

 1

 1

 1

 1

 1

 374

 374

 374

 374

 374

 544

 544

 544

 544

 544

 271

 271

 271

 271

 271

 160

 160

 160

 160

 160

 100

 100

 100

 100

 100

 31

 31

 31

 31

 31

 65

 65

 65

 51

 51

 14

 14

 11

 11

 11

 11

 10

 1

 7

 7

 7

 7

 7

 11

 6

 6

 6

 6

 2

 2

 2

 2

 2

 1

 1

 1

 1

 1

 1

 1

 1

 1

 1

 4

 4

 4

 4

 4

 3

 3

 3

 3

 3

 2

 2

 2

 2

 2

 2

 2

 2

 2

 2

 2

 2

 2

 2

 2

 3

 1

 1

 1

 1

 2

 2

 2

 2
