# Supplementary material for: The Bacterial Community Diversity of Bathroom Hot Tap Water Was Significantly Lower Than That of Cold Tap and Shower Water
Source: Front Microbiol. 2021 Apr 23;12:625324. doi: 10.3389/fmicb.2021.625324 (PMC8102780; doi:10.3389/fmicb.2021.625324)
Supplement: Supplementary file 1 [file Presentation_1.zip › Supplementary material for the Proof/Supplementary material.docx]

***Supplementary material for***

**The Bacterial Community Diversity of Bathroom Hot Tap Water
Was Significantly Lower Than That of Cold Tap and Shower Water**

**Chiqian Zhang^1†^, Ke Qin^2†^, Ian Struewing^3^, Helen Buse^3^, Jorge Santo Domingo^3^, Darren Lytle^3^, and Jingrang Lu^3*^**

Affiliations:

^1^Pegasus Technical Services, Inc., Cincinnati, Ohio, United States

^2^Oak Ridge Institute for Science and Education Participation Program, Office of Research and Development, United States Environmental Protection Agency, Cincinnati, Ohio, United States

^3^Office of Research and Development, United States Environmental Protection Agency, Cincinnati, Ohio, United States

**^*^Correspondence**: Jingrang Lu, [lu.jingrang@epa.gov](mailto:lu.jingrang@epa.gov)

^†^These authors have contributed equally to this work.

**Table S6**. Analysis of the Yue and Clayton theta and Jaccard distance matrices with PCoA and NMDS

| **Distance matrix** | ***R*^2^ for** | |
| --- | --- | --- |
|  | **PCoA** | **NMDS** |
| Yue and Clayton theta | 0.833 | 0.914 |
| Jaccard | 0.729 | 0.519 |

Analyzed with Mothur.

**Table S7**. Normality tests (the Shapiro-Wilk test) for water and ambient temperatures

| **Temperature** (°C) | **Statistic** | ***p*** |
| --- | --- | --- |
| Cold tap water-First draw | 0.927 | 0.222 |
| Cold tap water-Second draw | 0.943 | 0.422 |
| Hot tap water-First draw | 0.945 | 0.481 |
| Hot tap water-Second draw | 0.912 | 0.123 |
| Shower water-First draw | 0.950 | 0.491 |
| Shower water-Second draw | 0.967 | 0.781 |
| Ambient | 0.923 | 0.185 |

Analyzed with SPSS^®^.

**Table S10**. Phyla shared by cold tap, hot tap, and shower water

| **Phylum** | **Number of OTUs in each phylum** | **Relative abundance for each phylum (%)** |
| --- | --- | --- |
| Acidobacteria | 2 | 0.4 |
| Actinobacteria | 62 | 13.1 |
| Bacteria_unclassified | 2 | 0.4 |
| Bacteroidetes | 26 | 5.5 |
| Chlamydiae | 4 | 0.8 |
| Cyanobacteria | 23 | 4.9 |
| Dependentiae | 4 | 0.8 |
| Epsilonbacteraeota | 3 | 0.6 |
| Firmicutes | 19 | 4.0 |
| Gemmatimonadetes | 2 | 0.4 |
| Nitrospirae | 1 | 0.2 |
| Planctomycetes | 8 | 1.7 |
| Proteobacteria | 311 | 65.9 |
| Spirochaetes | 1 | 0.2 |
| Verrucomicrobia | 4 | 0.8 |
| Total | 472 | 100 |

Analysized with Mothur. We identified 15 unique phyla from the 472 OTUs shared by cold tap, hot tap, and shower water.

**Table S11**. Number of OTUs identified from groups of water samples

| **Group** | **Number of samples** | **Number of OTUs** |
| --- | --- | --- |
| Cold tap water-First draw | 16 | 1,086 |
| Cold tap water-Second draw | 16 | 1,356 |
| Cold tap water | 32 | 1,977 |
| Hot tap water-First draw | 16 | 874 |
| Hot tap water-Second draw | 16 | 950 |
| Hot tap water | 32 | 1,453 |
| Shower water-First draw | 16 | 1,251 |
| Shower water-Second draw | 16 | 1,272 |
| Shower water | 32 | 1,981 |
| All samples | 96 | 3,821 |

Analysized with Mothur.

**Table S12**. A multiple linear regression model predicting the Shannon and Inverse Simpson diversity indices of shower water with those of cold and hot tap water

| **Regression result** | **Shannon diversity index of shower water** | **Inverse Simpson diversity index of shower water** |
| --- | --- | --- |
| Coefficient of determination (*R*^2^) | 0.451 | 0.333 |
| Adjusted *R*^2^ | 0.413 | 0.286 |
| *F*-ratio (*p*) for the overall model | 11.889 (< 0.001^*^) | 7.223 (0.003^*^) |
| Unstandardized coefficient (*p*) of cold tap water | 0.766 (0.005^*^) | 0.917 (0.023^*^) |
| Unstandardized coefficient (*p*) of hot tap water | 0.470 (0.056) | 0.430 (0.276) |
| Unstandardized coefficient (*p*) of the constant | -0.231 (0.689) | 0.428 (0.844) |

Analyzed with SPSS^®^. ^*^: Statistical significance (*p* < 0.05).

**Table S13**. The AMOVA and HOMOVA tests on the Yue and Clayton theta distance matrix

| **Comparison of groups** | **AMOVA test** | | | |  | **HOMOVA test** | |
| --- | --- | --- | --- | --- | --- | --- | --- |
|  | ***F* ratio** | **DF Between groups** | **DF Within groups** | ***p*** |  | **Bartlett's statistic (*B* value)** | ***p*** |
| Cold tap water: First draw versus second draw | 2.211 | 1 | 30 | 0.064 |  | 0.563 | 0.231 |
| Hot tap water: First draw versus second draw | 4.547 | 1 | 30 | 0.016^*^ |  | 0.0306 | 0.743 |
| Shower water: First draw versus second draw | 0.164 | 1 | 30 | 0.957 |  | 0.0896 | 0.426 |
| Cold tap water versus Hot tap water versus Shower water | 6.049 | 2 | 93 | < 0.001^*^ |  | 1.222 | 0.072 |
| Cold tap water versus hot tap water | 7.766 | 1 | 62 | < 0.001^*^ |  | NA | NA |
| Cold tap water versus shower water | 1.042 | 1 | 62 | 0.373 |  | NA | NA |
| Hot tap water versus shower water | 9.911 | 1 | 62 | < 0.001^*^ |  | NA | NA |
| Cold tap water: March to July in 2012 versus March to July in 2013 | 5.878 | 1 | 16 | < 0.001^*^ |  | 1.981 | 0.012^*, ⁑^ |
| Hot tap water: March to July in 2012 versus March to July in 2013 | 10.931 | 1 | 16 | < 0.001^*^ |  | 0.751 | 0.100 |
| Shower water: March to July in 2012 versus March to July in 2013 | 10.959 | 1 | 16 | < 0.001^*^ |  | 0.395 | 0.154 |

Analyzed with Mothur. DF: Degree(s) of freedom. ^*^: Statistical significance (*p* < 0.05). ^⁑^: The variation of the bacterial community for the early cold tap water samples (0.074) was smaller than that for the late cold tap water samples (0.207).

**Figure S1**. Rarefaction curves for the first and second draws of cold tap, hot tap, and shower water. Mothur gave NA's when no more new OTUs were identified after sampling more contigs. We replaced the NA's with the maximum number of OTUs identified for each water sample. Afterward, we calculated the average number of OTUs per sample for the first and second draws to generate the rarefaction curves.

**Figure S2**. The Shannon diversity index as a function of (**A**) sampling date, (**B**) water temperature, and (**C**) total chlorine residual concentration for the first and second draws of cold tap, hot tap, and shower water.

**Figure S3**. The Inverse Simpson diversity index as a function of (**A**) sampling date, (**B**) water temperature, and (**C**) total chlorine residual concentration for the first and second draws of cold tap, hot tap, and shower water.

**Figure S4**. The linear correlation between ambient and cold tap water temperatures. The *p*-values for the overall model were calculated with SPSS^®^.

**Figure S5**. The linear correlation between free and total chlorine residual concentrations. The adjusted *R*^2^ value and *p*-value for the overall model were calculated with SPSS^®^.

**Figure S6**. The linear correlation between total chlorine residual concentration and water temperature. The *p*-value for the overall model was calculated with SPSS^®^.

**Figure S7**. The relative abundance of dominant bacterial phyla in the water samples. (**A**) Cold tap, hot tap, and shower water (three groups of water samples). We assigned a phylum to each contig for each water sample with Mothur and then calculated the relative abundance of each phylum for each group of samples. We displayed a phylum only if its relative abundance is greater than 0.50% in at least one group. (**B**), (**C**), (**D**), (**E**), (**F**), and (**G**): Changes in the relative abundance of dominant phyla over the 16-month sampling period for the first draw of cold tap water, second draw of cold tap water, first draw of hot tap water, second draw of hot tap water, first draw of shower water, and second draw of shower water, respectively. We assigned a phylum to each contig for each water sample with Mothur and then calculated the relative abundance of each phylum for each sample.

**Figure S8**. Changes in the relative abundance of dominant bacterial classes over the 16-month sampling period. (**A**), (**B**), (**C**), (**D**), (**E**), and (**F**): The first draw of cold tap water, second draw of cold tap water, first draw of hot tap water, second draw of hot tap water, first draw of shower water, and second draw of shower water, respectively. We assigned a class to each contig for each sample with Mothur and then calculated the relative abundance of each class for each sample.

**Figure S9**. The relative abundance of dominant bacterial families in cold tap, hot tap, and shower water (three groups of samples). We assigned a family to each contig for each water sample with Mothur and then calculated the relative abundance of each family for each group of samples. We displayed a family only if its relative abundance is greater than 5.00% in at least one group.

**Figure S11**. The Inverse Simpson diversity indices of cold tap, hot tap, and shower water as a function of water temperature.

**Figure S12**. The 3D PCoA of the Yue and Clayton theta distance matrix for all water samples. Percent in the title of an axis is the fraction of the total variation that can be explained by that axis. Plotted with a SigmaPlot software (version 14.0, Systat Software, Inc., San Jose, California, USA).

**Figure S13**. The 3D NMDS of the Yue and Clayton theta distance matrix for all water samples. Plotted with a SigmaPlot software (version 14.0, Systat Software, Inc., San Jose, California, USA).
